# Chemo-Biocascade Reactions Enabled by Metal–Organic Framework Micro-Nanoreactor

**DOI:** 10.34133/2022/9847698

**Published:** 2022-08-15

**Authors:** Jing Zhang, Yu Shen, Na Jin, Xiaopeng Zhao, Hongfeng Li, Ning Ji, Yingjie Li, Baoli Zha, Lin Li, Xikuang Yao, Suoying Zhang, Fengwei Huo, Weina Zhang

**Affiliations:** ^1^Key Laboratory of Flexible Electronics (KLOFE), Institute of Advanced Materials (IAM), Nanjing Tech University (NanjingTech), Nanjing 211800, China; ^2^The Institute of Flexible Electronics (IFE, Future Technologies), Xiamen University, Xiamen, 361005 Fujian, China

## Abstract

The one-pot combination of biocatalytic and chemocatalytic reactions represents an economically and ecologically attractive concept in the emerging cascade processes for manufacturing. The mutual incompatibility of biocatalysis and chemocatalysis, however, usually causes the deactivation of catalysts, the mismatching of reaction dynamic, and further challenges their integration into concurrent chemo-biocascades. Herein, we have developed a convenient strategy to construct versatile functional metal–organic framework micro-nanoreactors (MOF–MNRs), which can realize not only the encapsulation and protection of biocatalysts but also the controllable transmission of substances and the mutual communication of the incompatible chemo-biosystems. Importantly, the MOFs serving as the shell of MNRs have the capability of enriching the chemocatalysts on the surface and improving the activity of the chemocatalysts to sufficiently match the optimum aqueous reaction system of biocatalysts, which greatly increase the efficiency in the combined concurrent chemo-biocatalysis. Such strategy of constructing MOF–MNRs provides a unique platform for connecting the “two worlds” of chemocatalysis and biocatalysis.

## 1. Introduction

In the long course of the evolution, enzymes with features of high activity and substrate specificity have developed to perform a wide range of catalytic functions, and their cascade reactions in the cell provide a research basis for revealing the rule of biological activities [1]. Learning from nature, researchers have been pursuing the integration of two or more types of catalysts in vitro at the micro- and nanoscale levels for designing highly efficient hybrid catalysts [2]. While achieving their biological functions in multienzyme catalytic cascades [3–5], enzymes can, when compatible with chemocatalysts, possess high activity and enantioselectivity under certain conditions [6, 7]. In comparison to the enzyme cascades, chemocatalysts with a range of new advantages may enrich the enzymes' repertoire, such as good stability, various substrate choices, and diverse reaction types [8]. However, the reaction conditions of biocatalysts are usually different from those of the chemocatalysts in that the two types of catalysts are frequently deactivated in the one-pot reaction process [6]. Generally speaking, some biocatalysts (enzymes) prefer the aqueous phase reaction system at relatively low temperature [9, 10], whereas the chemocatalysts favor the organic phase at high temperature [11]. Also, the matching between chemical catalysts' and biocatalysts' activity is also a challenging issue [12, 13]. Therefore, how to achieve compatibility between the two catalysts becomes a major concern in the one-pot integration of biocatalysis and chemocatalysis.

As early as 1980, Makkee et al. [14] reported the first chemo-biocatalysis cascade system in two separated reaction units. They used heterogeneous metal catalysts to achieve hydrogenation reaction and then combined with enzymes to implement isomerization reaction. At that time, the conversion of D-glucose to D-mannitol not only promoted the development of sugar manufacturing but also provided a new method for combining chemo- and biocatalysts to achieve unique chemical conversion. However, the two-step cascade system faced problems of low efficiency, separation, and purification, which motivated the researchers to develop a one-pot cascade system. Generally, there are three ways to realize the one-pot chemo-biocatalysis efficiently. First is improving the activity of the chemocatalysts under the condition of enzyme reaction. Li et al. synthesized lipase-palladium (Pd) nanohybrids by using single lipase-polymer nanoconjugates as confined nanoreactors [15], in which the Pd nanoparticle (Pd NP) size was down to 0.8 nm. The 0.8 nm Pd NPs not only exhibited enhanced activity in racemization of (S)-1-phenylethylamine ((S)-1-PEA) but also matched efficient reaction dynamics of lipase at low temperature and aqueous solutions. Therefore, reducing the size of metal nanoparticles or introducing active supports in the enzyme reaction system can improve the efficiency of the overall cascade reaction. Second is enhancing the enzyme activity and stability through gene regulation or chemical modification [16]. A typical example is enhanced activation of laccase, and carbonic anhydrase was observed when Cu_3_(PO_4_)_2_·3H_2_O nanoflowers were incorporated into the enzyme solution [17]. The activity of the enzymes in Cu_3_(PO_4_)_2_·3H_2_O nanoflowers could largely expand the catalytic range and boost the efficiency of enzymes. Although the above two types of approaches have rendered the chemo- or biocatalysts compatibility to a certain extent, they still cannot completely avoid the mutual deactivation due to the direct contact between the two catalysts in the one-pot reaction system, achieving the compatibility and activity matching in the chemo-bioconcurrent system. Subsequently, the strategies of micro-nanoreactors (MNRs) were developed to realize the spatial separation of chemo- and biocatalysts to prevent mutual deactivation. Numerous materials like phospholipid bilayers [18], polymers [19], and mesoporous organosilicons [20] were assembled into MNRs, such as liposomes, polymersomes, and colloidosomes, for the encapsulation of chemo- or biocatalysts [21]. For instance, mesoporous core–shell structured nanocatalysts with PdPt bimetallic core and enzyme-immobilized polydopamine (PDA) shell were designed by Gao et al. [22]. The concept of integrating metals and enzymes at spatially distinct locations of core–shell materials has great potential in performing efficient one-pot cascade reactions. However, some major challenges remain in the field of concurrent chemo-biocascades under the conditions of low temperatures and aqueous solution by MNR strategy: (1) to achieve selective transport of reaction substrates by shell of MNRs to adapt to more complex reaction system, (2) to improve the stability and activity of the enzyme, and (3) to enhance catalytic activity of the chemocatalysts that can match the enzymatic reactions at ambient conditions. Hence, the development of a suitable material for constructing MNRs becomes key to achieve efficient chemo- and biocatalysis in one-pot.

**Figure 1:**
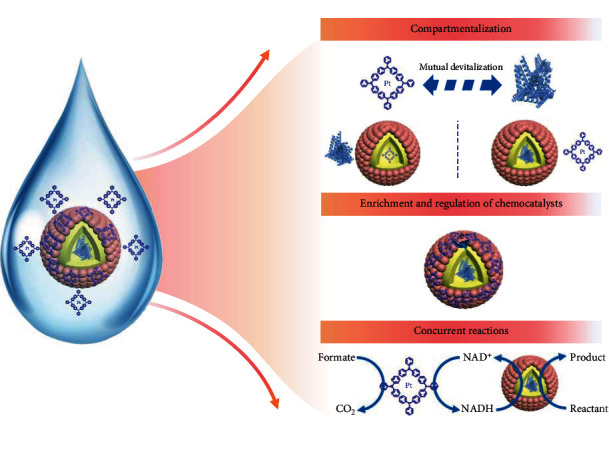
Schematic representation of AlcDH/NAD^+^@MOF–MNRs combined with metal complex. The enzymes are protected via the size-sieving effect of MOF–MNRs to avoid inactivation by the metal complex. The chemical microenvironments of MOFs can enrich chemocatalysts and regulate their electronic status. The overall reaction is driven by a “sacrifice reaction” of formate to carbon dioxide (CO_2_), and then, through the mediation of NAD^+^ and NADH, reactants can undergo asymmetric reactions to become target products. AlcDH: alcohol dehydrogenase; NAD^+^: nicotinamide adenine dinucleotide.

Metal–organic frameworks (MOFs) [23–27] are a kind of organic–inorganic hybrid porous materials with a periodic network structure, which are extensively used in gas storage [28–31], catalysis [32–36], separation [37], and medicine [38–41]. Due to adjustable pores and uniform pore sizes [42], MOFs as the shell of MNRs will not only enable the separation of chemo- and biocatalysts but also precisely regulate the transport of substances [13]. Moreover, the organic–inorganic hybrid system of MOFs with abundant physical–chemical microenvironment can regulate the catalytic activity of chemo- and biocatalysts [4, 43]. Therefore, MOFs as the constructing units of MNRs may promise the concurrent chemo-biocascades. Herein, we develop a facile strategy to construct metal–organic framework micro-nanoreactors (MOF–MNRs) by self-assembling MOF particles on oil–water surface, which can realize efficient encapsulations for chemo- or biocatalysts, selective transmission for substrates, enhanced activity for chemocatalysts, and mutual communications for incompatible chemo-biocatalysts in concurrent chemo-biocascades (Figure 1). In contrast to previous reports on MNRs constructed by other materials, MNRs with MOFs as the shell have achieved controllable communications of reaction substrates and size selectivity of the chemocatalysts, ensuring their efficiency in complex reaction environments. The chemical microenvironments of MOFs can enrich chemocatalysts and regulate their electronic status, further boosting their activity and adapting to the reaction condition of enzymes at a relatively low temperature and aqueous solution. Notably, the as-prepared hybrid chemo-biocatalysts (AlcDH/NAD^+^@MOF–MNRs combined with Pt[(C_6_H_5_)_3_P]_4_) exhibit an improved efficiency as well as good enantioselectivity in concurrent chemo-biocatalysis.

## 2. Results

### 2.1. Synthesis and Structure Characterization of MOF–MNRs

**Figure 2:**
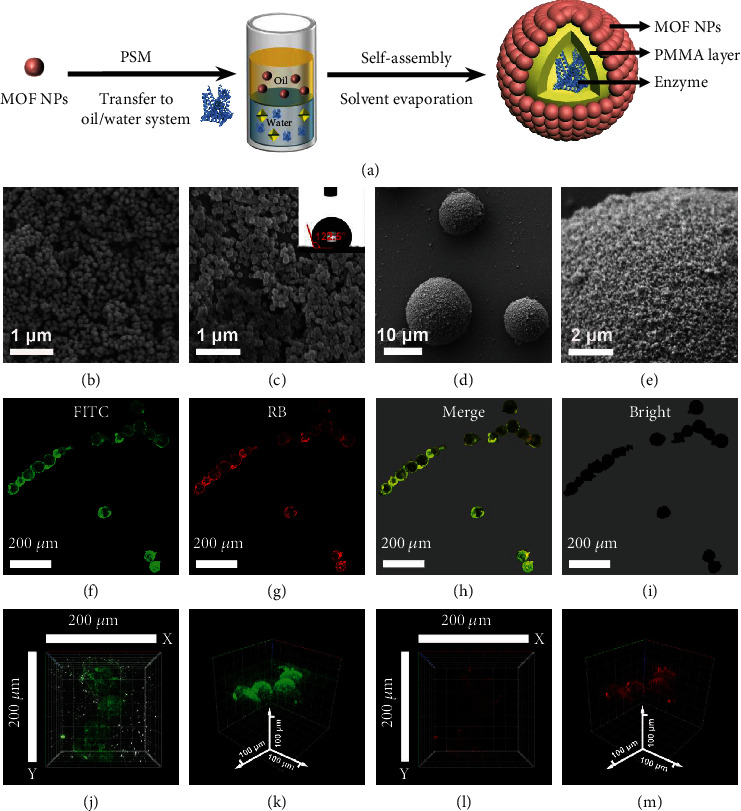
Assembly process and characterization of MOF–MNRs. (a) Systematic illustration of the formation process of MOF–MNRs. PSM: postsynthetic modification. (b) SEM image of MOF NPs. (c) SEM image and contact angle of hydrophobic modified MOF NPs. (d) SEM image of the overall view of MOF–MNRs. (e) SEM image of the surface layer of MOF–MNRs. (f–i) Laser scanning confocal microscope images of FITC (*λ*_ex_ = 488 nm) modified AlcDH and RB (*λ*_ex_ = 543 nm) modified NAD^+^, which are both encapsulated in the MOF–MNRs. (j–m) *z*-axis scan 3D fluorescence stacking chart at different excitation wavelengths.

To verify the capability of MOF–MNRs in concurrent chemo-biocascade catalysis, UiO-66-NH_2_, one kind of MOFs, is taken as an example to construct the MOF–MNRs mainly due to its stable frame structure and easy modification. The formation process of MOF–MNRs is demonstrated in Figure 2(a). UiO-66-NH_2_ NPs are synthesized through the reaction among zirconium chloride (ZrCl_4_), benzoic acid (C_7_H_6_O_2_), and 2-aminoterephthalic acid (NH_2_-BDC) mixture in N, N-dimethylformamide (DMF) at 120°C for 24 h. The prepared UiO-66-NH_2_ NPs are well dispersed and possess uniform size of ~100 nm (Figure 2(b)). The postmodification of heptanoic anhydride is employed to regulate the hydrophobicity of UiO-66-NH_2_ NPs, in which the acid anhydride bond of heptanoic anhydride is amidated with the exposed amino group on the NH_2_-BDC ligand and a heptanoic acid is grafted on the frameworks of UiO-66-NH_2_. In this way, the originally hydrophilic UiO-66-NH_2_ NPs become hydrophobic ones (the contact angle is 122.5°) mainly because the alkyl chain of heptanoic acid substitutes the amino group of ligands (Figure 2(c)). The size and morphology of UiO-66-NH_2_ NPs reveal no obvious change after the hydrophobic modification. The increase of contact angle and the decrease in gravimetric Brunauer–Emmett–Teller (BET) surface areas of the hydrophobic UiO-66-NH_2_ NPs further indicate the successful hydrophobic modification (Figure S1), which facilitates the subsequent assembly of modified MOF NPs in water-in-oil Pickering emulsions. MOF–MNRs are fabricated by self-assembly of the hydrophobic UiO-66-NH_2_ on water–oil interface, with polymer precipitation being used as the support layer for enhancing stability. The spherical MOF–MNRs with a dense MOF layer on the surface were about 10-20 *μ*m in diameter (Figures 2(d) and 2(e)). MOF–MNRs presented similar crystal structure to the UiO-66-NH_2_ NPs, as demonstrated by the X-ray diffraction (XRD) patterns (Figure S2). Based on the above characterizations, it can be concluded that MOF particles are self-assembled on the surface of MNRs and form a dense shell. Therefore, MOF–MNRs not only own the MOF shell for potential selective transport of molecules but also possess a hollow cavity for possibly encapsulating chemo- or biocatalysts and providing reaction place (Figure S3).

MOF–MNRs can be acquired by self-assembly of the hydrophobic MOF NPs on water–oil interface, supported by a polymer layer to enhance stability. The dispersion of hydrophobic MOF NPs in the oil phase and the addition of the hydrophilic enzymes in the water phase can encapsulate the enzymes inside the MOF–MNRs through the self-assembly on Pickering emulsion interface, contributing to the spatial separation of chemo- and biocatalysts in one-pot cascade reactions. Here, a zinc-containing metalloenzyme (alcohol dehydrogenase (AlcDH)) with broad substrate specificity and its cofactors (nicotinamide adenine dinucleotide (NAD^+^)) is chosen to study the encapsulation process of enzymes. The fluorescence lifetime imaging microscope (FLIM) of laser scanning confocal microscope is used to measure and analyze the distribution of enzyme in MOF–MNRs. For fluorescein isothiocyanate isomer (FITC) labeled AlcDH and rhodamine B (RB) labeled NAD^+^ in the MOF–MNRs, their fluorescence microscope transmission images (Figures 2(f)–2(i)) and the *z*-axis stacked 3D fluorescence images (Figures 2(j)–2(m)) show strong fluorescence intensity, corresponding to green light and red light, respectively, evidencing the existence of both AlcDH and NAD^+^ in the MOF–MNRs. In order to confirm the location of enzyme in MOF–MNRs, the stepping method of scanning fluorescence process is employed, and the fluorescence intensity is observed from weak to strong and again to weak along the *z*-axis of MOF–MNRs (Figure S4). The above results reflect the even distribution of enzyme in the three-dimensional space of the cavity, indicating that the enzymes are successfully encapsulated in MOF–MNRs.

### 2.2. Multifunctionality of MOF–MNRs

**Figure 3:**
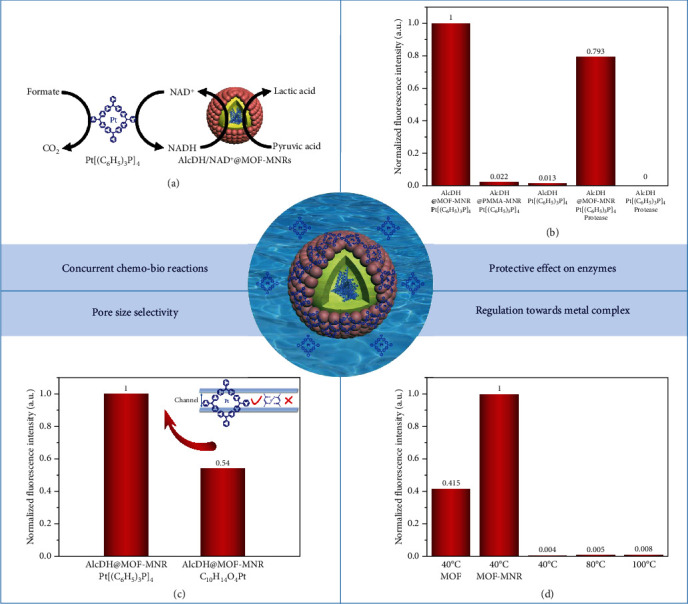
MOF–MNRs can efficiently encapsulate chemo- or biocatalysts, realizing mutual communications for incompatible chemo-biocatalysts, achieving selective transmission for substrates, and enhancing the activity for chemocatalysts in concurrent chemo-biocascades. (a) Schematic illustration about reduction of pyruvic acid to lactic acid by the concurrent chemo-bioreaction of Pt[(C_6_H_5_)_3_P]_4_–AlcDH/NAD^+^@MOF–MNRs. (b) Fluorescence illustration of the concurrent reactions' catalytic activity, which is demonstrated by the intensity of NADH generated under different microenvironments, showing the protective effect of MOF–MNRs on AlcDH. (c) Fluorescence assay of the lactic acid catalyzed by metal complexes with different sizes, illustrating the size selectivity of MOF layer. (d) Illustration of the optimization of the reaction activity of chemocatalysts by MOF–MNRs.

Homogeneous metal catalysis combined with enzymes has historically dominated the chemical and pharmaceutical industries especially by the use of transitional metals as chemocatalyst components. The compatibility of both catalysts and reaction conditions becomes critical when simultaneously proceeding transition metals and enzyme reactions in concurrent chemo-biocascades [6, 44–46]. Here, the compatible capability of the MOF–MNRs in concurrent chemo-biocatalysis is further demonstrated, through combination of the metal complex catalyst (tetrakis(triphenylphosphine)platinum (Pt[(C_6_H_5_)_3_P]_4_)), the regeneration of the coenzyme NAD^+^ with NADH–AlcDH reduction of pyruvic acid for synthesis of lactic acid (Figure 3(a)). And we adopt a widely applicable method to investigate the pyruvate reduction reaction at a certain amount of time by measuring the intensity of NADH [47]. It is well acknowledged that the reduction process cannot occur if there is only AlcDH or Pt[(C_6_H_5_)_3_P]_4_ serving as catalysts in the reaction system due to the reaction nature of concurrent chemo-biocatalysis (Figure S5). When directly mixing the AlcDH and Pt[(C_6_H_5_)_3_P]_4_ in a reaction system, AlcDH will be deactivated (Figure 3(b)). The successful encapsulation of enzyme in MOF–MNRs makes compartmentalization of the chemo- and biocatalysts come true, and then, the catalysts consisting of AlcDH/NAD^+^@UiO-66-NH_2_-MNRs and Pt[(C_6_H_5_)_3_P]_4_ exhibit good conversion in the reduction of pyruvic acid to lactic acid at 40°C and 200 rpm in aqueous solution for 12 h. The results mainly contribute to that the uniform micropore size of UiO-66-NH_2_ (pore size: 6 Å) [48] discourages the entrance of the Pt[(C_6_H_5_)_3_P]_4_ to contact with AlcDH. Furthermore, AlcDH/NAD^+^@UiO-66-NH_2_-MNRs show good recyclability in 3 cycles of concurrent cascade chemo-biocatalysis (Figure S6). To further investigate the compartmentalization effect of MOF–MNR structure, the polymer (polymethyl methacrylate (PMMA)) micro-nanoreactors (PMMA–MNRs) are chosen as a comparison. PMMA–MNRs with spherical shapes are obtained by following the similar procedure expect that MOF NPs are replaced by Span 80 (Figure S7). Although PMMA–MNRs can achieve the encapsulation of enzyme, AlcDH/NAD^+^@PMMA–MNRs and Pt[(C_6_H_5_)_3_P]_4_ catalysts exhibit lower activity (Figure S8), which is caused by the nonuniform meso/macropores in PMMA structure with no molecular sieving ability [42]. The diffusion comparison of MOF–MNRs and PMMA–MNRs clearly shows their differences in protecting biomolecules (Figure S9). Besides encapsulating enzymes, our strategy can also encapsulate chemocatalysts using the oil-in-water Pickering emulsion system, and thus, space separation can be achieved. Although Pt[(C_6_H_5_)_3_P]_4_@MOF–MNRs with AlcDH/NAD^+^ demonstrate good conversion in the reduction of pyruvic acid to lactic acid when there are substances such as proteases in the system, the enzymes outside the cavity tend to be inactivated (Figure S10).

In addition, MOF–MNRs can effectively improve the stability of the encapsulated enzymes. AlcDH/NAD^+^@UiO-66-NH_2_-MNRs and pure AlcDH are dispersed in phosphate buffer at room temperature for several days, and the UV-vis spectra are employed to analyze the activity of the AlcDH. The enzyme activity was further evaluated after the reaction in the systems of pure AlcDH, AlcDH/NAD^+^@PMMA–MNRs, and AlcDH/NAD^+^@MOF–MNRs. The intensity of special peak in 260~278 nm decreases gradually, indicating decreased AlcDH activity with the change of encapsulated shell material from UiO-66-NH_2_-MNRs, PMMA–MNRs to pure enzymes (Figures S11–S13). One week later, AlcDH/NAD^+^@UiO-66-NH_2_-MNRs combined with Pt[(C_6_H_5_)_3_P]_4_still remain comparable conversion in the reduction of pyruvic acid to lactic acid, in contrast to the activity of the fresh AlcDH/NAD^+^@UiO-66-NH_2_-MNR system (Figure S14). The slight decrease in activity might be attributed to the falling of some UiO-66-NH_2_ particles from the surface of MNRs, as confirmed by the SEM images, in which the smooth surface of the inner polymer layer can be observed (Figure S15). Therefore, MOF–MNRs present an enhanced barrier with a good protective effect on AlcDH [4, 48, 49], indicating their great potential in effective encapsulation and separation protection. The other feature of MOF–MNRs is that certain MOFs with uniform micropores can control the molecular transport, in which only the molecules with smaller size than aperture size of MOFs can enter and contact with the enzyme while the bigger size molecules can not. Hence, one kind of metal complex catalysts with smaller size, platinum acetylacetonate (C_10_H_14_O_4_Pt) rather than Pt[(C_6_H_5_)_3_P]_4_, was used to demonstrate the selective transport of MOF channel. When AlcDH/NAD^+^@UiO-66-NH_2_-MNRs are used as a biocatalyst, the conversion of concurrent chemo-biocatalysis decreases gradually after introducing smaller size C_10_H_14_O_4_Pt as chemocatalysts (Figure 3(c)). The MOFs as shell of MNRs can allow small-sized of metal complex catalysts to enter the inside of the MOF–MNRs as well as to denature the enzyme. The designed MOF–MNRs can not only protect enzyme against the metal catalyst deactivation but also ensure the occurrence of the concurrent chemo-biocascade catalysis in more complex reaction system, such as in the one with protease and metal catalysts coexisting. Protease, one kind of enzyme, can induce the proteolysis of some enzymes (e.g., AlcDH) and decrease enzymatic activity. The AlcDH/NAD^+^@UiO-66-NH_2_-MNRs are highly resistant to digestion by protease and retain the initial activity of AlcDH in concurrent chemo-biocatalysis, mainly due to the uniform micropore structure of UiO-66-NH_2_ (Figure S10). In the concurrent chemo-biocatalysis, MOF–MNRs not only demonstrate compartmentalization effect by the encapsulation and protection of biocatalysts but also display size selectivity of the substances and shorten the reaction distance and the mutual communication of the chemo-bioincompatible catalysts.

### 2.3. Enrichment and Regulation of Chemocatalysts by MOF–MNRs

**Figure 4:**
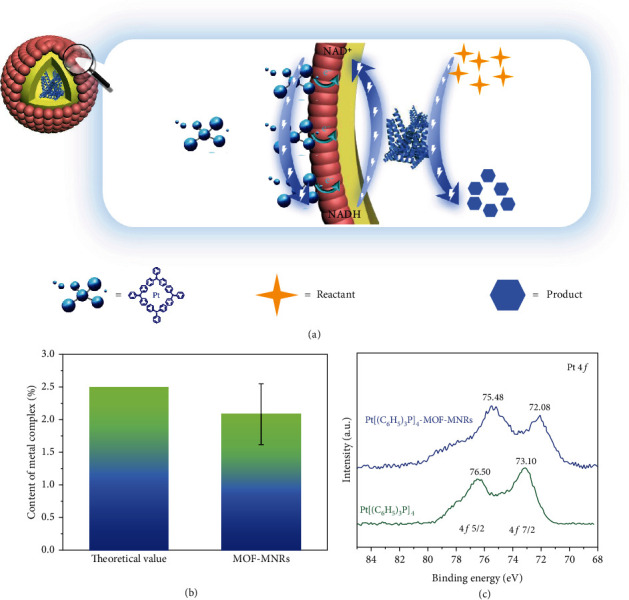
The chemical microenvironments of MOFs can enrich chemocatalysts and regulate their electronic status. (a) Schematic illustration about electrostatic interaction between MOF–MNRs and Pt[(C_6_H_5_)_3_P]_4_. (b) ICP analysis of the content of metal complex on the surface of the MOF–MNRs. (c) Pt 4*f* spectrum of Pt[(C_6_H_5_)_3_P]_4_–MOF–MNRs and Pt[(C_6_H_5_)_3_P]_4_.

Generally, most enzymes prefer an aqueous solution at ambient temperature, whereas the same environment will restrain chemocatalysts from showing good activity. Fortunately, the physiochemical microenvironment of MOFs can optimize the reaction activity of chemocatalysts by tuning the electronic state or confinement effect [50–53], such as metal NPs and organic catalysts. Hence, some control experiments are designed to demonstrate the capability of UiO-66-NH_2_-MNRs to improve the activity of metal complex catalysts (Pt[(C_6_H_5_)_3_P]_4_). During the partial reactions of hydrogenation of NAD^+^ to NADH by Pt[(C_6_H_5_)_3_P]_4_, the conversion gradually increases along with the reaction temperature rising from 40°C to 100°C, which corresponds to the common phenomena of high activity of chemocatalysts at high temperatures (Figure 3(d)). Interestingly, Pt[(C_6_H_5_)_3_P]_4_–MOF–MNR displays extremely high catalytic efficiency at the optimum reaction condition (40°C and PBS solution) of AlcDH compared to bare Pt[(C_6_H_5_)_3_P]_4_ at 100°C. Two main reasons are deduced as follows: (1) the amount of Pt[(C_6_H_5_)_3_P]_4_ on the surface of UiO-66-NH_2_ tends to be increased due to the electrostatic interaction (Figure 4(a)), as confirmed by the zeta potential (Table S1) and SEM mapping images (Figure S16). The content of metal complex on the surface of the MOF–MNRs is about 2% (Figure 4(b)). Such enrichment facilitates reaction acceleration; (2) the electronic state of Pt[(C_6_H_5_)_3_P]_4_ is regulated by the interaction of organic ligands of UiO-66-NH_2_ and Pt[(C_6_H_5_)_3_P]_4_, which is demonstrated by the X-ray photoelectron spectroscopy (XPS) analysis. In the Pt 4*f* spectrum, the two peaks of pristine Pt[(C_6_H_5_)_3_P]_4_ at 73.10 and 76.50 eV were corresponded to Pt 4*f* 7/2 and 4*f* 5/2, respectively, whereas shift to a lower binding energy after being adsorbed on MOF–MNRs (Figure 4(c) and S17), indicating a partial transfer of electrons from UiO-66-NH_2_ to Pt[(C_6_H_5_)_3_P]_4_. The electron-rich status of Pt[(C_6_H_5_)_3_P]_4_ adsorbed on MOF–MNRs is probably the explanation for the higher activity of Pt[(C_6_H_5_)_3_P]_4_–MOF–MNRs than pure Pt[(C_6_H_5_)_3_P]_4_ under mild reaction conditions of biocatalysis. The results suggest that the increased rate of chemical reactions matches the reaction rate of enzymes, improving the overall chemo-bioreactions. Therefore, MOF–MNRs play a crucial role in developing highly efficient, concurrent chemo-biocatalysis.

### 2.4. MOF–MNR for Asymmetric Reduction

To verify that the enzymes encapsulated within MNR retain their chiral catalytic property, we further explored the asymmetric hydrogenation of benzyl acetone. Specifically, the Pt[(C_6_H_5_)_3_P]_4_ catalyzes the transfer hydrogenation of NAD^+^ to NADH in the presence of formate as a hydride donor. AlcDH catalyzes the asymmetric hydrogenation of benzyl acetone in the presence of NADH. The asymmetric hydrogenation of benzyl acetone is promoted by a mixture of Pt[(C_6_H_5_)_3_P]_4_–AlcDH/NAD^+^@MOF–MNRs in conjunction with NAD^+^ and formate (Table 1). The optimized reaction condition is in the PBS solution at 40°C for higher or lower temperatures would affect the activity of AlcDH. The products of (*S*) 4-phenyl-2-butanol are observed to attain 94.7% conversion and 87.8% ee after 20 h at 40°C by UiO-66-NH_2_-MNRs combining the Pt[(C_6_H_5_)_3_P]_4_ and AlcDH–NAD^+^. By contrast, the conversion is only 5.56% by directly mixing the Pt[(C_6_H_5_)_3_P]_4_ and AlcDH–NAD^+^ due to mutual deactivation of chemo-biocatalysts. Meanwhile, the combination of AlcDH/NAD^+^@PMMA–MNRs and Pt[(C_6_H_5_)_3_P]_4_ as catalysts results in low conversion of 65.40% and high 97.25% ee, since PMMA as shell could not entirely block the entrance of the metal complex catalysts. The MOF–MNRs' features of compartmentalization effect, protection of enzyme, regulation of the chemocatalysts, and communication of substrates can improve the synergistic catalytic ability in the concurrent chemo-biocatalysis for producing asymmetric (*S*) 4-phenyl-2-butanol. Such strategy has the potential to be extended to other one-pot reactions under mild conditions for achieving compatibility between metal complex catalysts and enzymes.

## 3. Conclusions

The designed MOF–MNRs can regulate the compatibility of chemo- and biocatalysts with high catalytic activity in concurrent chemo-biocatalysis. Firstly, different kinds of enzymes can be encapsulated inside MOF–MNRs by assembling on the oil–water interface for avoiding the external interference of metal complex catalysts or even protease enzyme, and their activity can be improved by the chemical microenvironment of MOF–MNRs. Secondly, MOF–MNRs have achieved size selectivity of the chemocatalysts and controllable communications of reaction substrates that originate from well–defined micropore nature of MOFs. Thirdly, the activity of chemocatalysts (Pt[(C_6_H_5_)_3_P]_4_) is improved by the enrichment of Pt[(C_6_H_5_)_3_P]_4_ and regulation of electronic status on MOF–MNR surface, which can adapt to the reaction condition of enzymes at a relatively low temperature and aqueous solution. The design of combining AlcDH/NAD^+^@MOF–MNRs and Pt[(C_6_H_5_)_3_P]_4_ catalysts results in an increased efficiency in concurrent chemo-biocatalysis and good enantioselectivity during the asymmetric transfer hydrogenation process. We believe that such study can provide promising research avenues in concurrent chemo-biocatalysis, which is not limited to the combination of biocatalyst and homogeneous chemocatalyst but can be extended to the integration of biocatalysts with heterogeneous chemocatalysts.

## 4. Materials and Methods

### 4.1. Materials and Measurements

ZrCl_4_, NH_2_BDC, N,N-dimethylformamide (DMF), alcohol dehydrogenase (AlcDH), nicotinamide adenine dinucleotide (NAD^+^), dodecane, protease, Span 80, metal complexes, and pyruvic acid were purchased from Sigma-Aldrich; benzoic acid and heptanoic anhydride were purchased from Tokyo Chemical Industry (TCI); 4-phenyl-2-butanol and benzyl acetone were purchased from Alfa; sodium formate was purchased from Macklin. These commercial reagents were used as received. Ultrapure water was obtained by our lab's ultrapure water machine (MZY-UR10V). SEM images were taken under 5 kV accelerating voltage using JEOL JSM-7600. Hitachi F-4600 fluorescence spectrometer was used to measure fluorescence data. UV absorption spectra were measured by SHIMADZU UV-1750. Hydrophobic modification data were carried out on a contact angle meter (KRUSS GmbH, DSA1005). The adsorption and desorption isotherms of nitrogen were analyzed by Brunauer–Emmett–Teller (BET). XRD patterns were recorded on a Rigaku (SC-XRD, XtaLAB mini II). Confocal fluorescence microscope data were carried out on ZEISS LSM880 confocal microscope system. The catalytic tests were analyzed by a high-performance liquid chromatography (HPLC) LC-20D.

### 4.2. Synthesis of UiO-66-NH_2_

UiO-66-NH_2_ NPs were prepared on the basis of Schaate's method [54] after revision. Firstly, ZrCl_4_ (0.16 g, 0.68 mmol), 2-aminoterephthalic acid (0.124 g, 0.68 mmol), and benzoic acid (0.84 g, 6.86 mmol) were mixed in 40 mL DMF. After that, dissolve the solution in a glass vial and sonicate for 10 min to make it evenly dispersed, followed by putting it in an oven at 120°C to react for 24 h. Next, it was cooled to room temperature, centrifuged, and washed 6 times with DMF and ethanol and centrifuged and put into vacuum oven.

### 4.3. Posthydrophobic Modification of UiO-66-NH_2_

We referred to Lohse et al.'s method for posthydrophobic processes [55]. After grinding UiO-66-NH_2_ into powder, 100 mg of them was dispersed into 10 mL heptanoic anhydride. The sample was sonicated for 15 min and then stirred at 100°C for 15 min. After cooling to room temperature and being centrifuged at 10000 rpm for 5 min, it was washed with ethanol for 3 times and put into vacuum oven at 120°C.

### 4.4. Preparation of MOF–MNRs and PMMA–MNRs

Briefly, MOF–MNRs were self-assembled from Pickering emulsions of water and oil phases by shear forces. The hydrophobically modified UiO-66-NH_2_ NPs were dissolved in the oil phase of 4 mL dodecane, and 15 mg PMMA was dissolved in acetone and various substances in the aqueous phase. After that, the emulsion was sheared using a PowerGen 125 set Homogenizer Polytron homogenizer (T-701FBT10P) fitted with an 8 mm dispersing tool at 18000 rpm. At last, the MOF–MNRs were obtained by removing the acetone using rotary evaporator under room temperature. For PMMA–MNRs, the surfactant Span 80 (0.5 wt%) was dispersed in the oil phase (4 mL), PMMA in the water phase of acetone, and PMMA–MNRs were formed under the action of shearing force.

### 4.5. Encapsulation of AlcDH and NAD^+^ inside MOF–MNRs

During the preparation of MOF–MNRs, 10 mg of AlcDH (99 × 69 × 58 Å, Figure S18) and 10 mg of NAD^+^ were dissolved in 100 *μ*L of 0.1 M phosphate buffer solution (PBS), and 32 *μ*L of each was added to 448 *μ*L of PMMA solution. After being shaken well and added to 4 mL of dodecane dispersion of UiO-66-NH_2_, the MOF–MNRs with water-in-oil structure could be formed under the action of shear force.

### 4.6. Confocal Fluorescence Microscope Characterization of MOF–MNRs

AlcDH was labelled by FITC; NAD^+^ was labelled by RB under dark conditions. After the labeling, the enzyme and NAD^+^ were coated according to the method in 4.4 and centrifuged and washed with 50% ethanol for 6 times, and then, the liquid was dropped in a confocal dish for further use. Afterwards, fluorescence microscopy and *z*-axis scanning were performed at 488 nm and 543 nm.

### 4.7. Catalytic Performances of AlcDH/NAD^+^@MOF–MNRs in the Reduction of Pyruvic Acid to Lactic Acid

The obtained AlcDH/NAD^+^@MOF–MNRs were transferred into PBS solution (2 mL, 0.1 M). Then, after the addition of metal complex (Pt[(C_6_H_5_)_3_P]_4_ (16 × 15 × 16 Å) or C_10_H_14_O_4_Pt (6 × 7 × 12 Å), Figure S18) (0.8 *μ*mol), formate (0.1 M), and pyruvic acid (2 M), the whole system was put into thermostatic shaker at 40°C for 12 h. Subsequently, after centrifugation and cell crusher treatment, the reaction efficiency of AlcDH/NAD^+^@MOF–MNRs with metal complex in the reduction of pyruvic acid was later determined by a previously reported method, in which the extent of the reaction proceeds and the production of pyruvate is investigated by the intensity of the reduced coenzyme NADH.

### 4.8. Diffusion Investigation of MOF–MNRs and PMMA–MNRs

The obtained FITC labelled AlcDH/NAD^+^@PMMA–MNRs and AlcDH/NAD^+^@MOF–MNRs were dispersed separately in 2 mL PBS (0.1 M). Then, the fluorescence intensity of each supernatant was measured every two hours for the next 12 hours.

### 4.9. Catalytic Tests of AlcDH/NAD^+^@MOF–MNRs in Asymmetric Hydrogenation Reaction

Substrate (benzyl acetone, 100 *μ*L), metal complex (0.8 *μ*mol), formate (0.1 M), and AlcDH/NAD^+^@MOF–MNRs (20 mg) were added to PBS solution (2 mL, 0.1 M). The reaction was carried out at 40°C for 20 h with stirring. The reaction mixture was collected and extracted with ethyl acetate (1 mL) for 3 times. Then, ethyl acetate was removed by rotary evaporation, and 1 mL ethanol was added for subsequent HPLC analysis.

## Figures and Tables

**Table 1:** Conversions of 4-phenyl-2-butanone in different reaction media for 20 h.

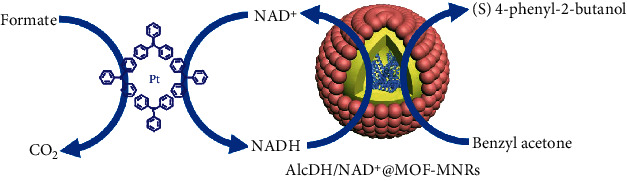			
Catalysts	Temperature (°C)	Conversion (%)	ee (%)
AlcDH/NAD^+^+Pt[(C_6_H_5_)_3_P]_4_	40	5.56	—
AlcDH/NAD^+^@MOF–MNRs+Pt[(C_6_H_5_)_3_P]_4_	20	70.70	94.30
AlcDH/NAD^+^@MOF–MNRs+Pt[(C_6_H_5_)_3_P]_4_	40	94.70	87.80
AlcDH/NAD^+^@MOF–MNRs+Pt[(C_6_H_5_)_3_P]_4_	60	0	—
AlcDH/NAD^+^@PMMA–MNRs+Pt[(C_6_H_5_)_3_P]_4_	40	65.40	97.25

## Data Availability

All data needed to evaluate the conclusions in the paper are present in the paper and the Supplementary Materials. Additional data related to this paper may be requested from the authors.

## Supplementary Materials

Supplementary Materials Supplementary 1. Figure S1: BET comparison of UiO-66-NH_2_ before and after hydrophobic modification. Supplementary 2. Figure S2: X-ray diffraction data for the synthesized, modified, and assembled MOF–MNRs. Supplementary 3. Figure S3: SEM images of MOF–MNRs. Supplementary 4. Figure S4: Fluorescence images of MOF–MNRs entrapped with FITC labelled AlcDH and RB labeled NAD+. Supplementary 5. Figure S5: comparison of the catalytic efficiency of the whole cycle reaction and the two half reaction catalyzed by either metal complex or AlcDH. Supplementary 6. Recyclability of AlcDH/NAD + @MOF–MNRs for concurrent chemo-biocatalysis in the reduction of pyruvic acid. Supplementary 7. Figure S7: SEM images of PMMA–MNRs obtained in the presence of surfactants. Supplementary 8. Figure S8: comparison of enzyme activity of AlcDH/NAD + @MOF–MNRs and AlcDH/NAD + @PMMA–MNRs after 12 h. Supplementary 9. Figure S9: Diffusion investigation of FITC labelled AlcDH inside MOF–MNRs and PMMA–MNRs. Supplementary 10. Figure S10: comparison of the fluorescence intensity of the generated NADH after exchanging the location of AlcDH and metal complex and in the presence of protease. Supplementary 11. Figure S11: comparison of the UV/Vis absorption of AlcDH between unencapsulated AlcDH/NAD+, AlcDH/NAD + @PMMA–MNRs, and AlcDH/NAD + @MOF–MNRs after the reactions. Supplementary 12. Figure S12: UV/Vis absorption of pure AlcDH within 50 h. Supplementary 13. Figure S13: UV/Vis absorption of AlcDH/NAD + @MOF–MNRs within 8 d. Supplementary 14. Figure S14: activities of the concurrent chemo-bioreactions of pyruvic acid to lactic acid immediately after fresh preparation of the catalysts (red line) and after storage together at room temperature for a time interval of one week (black line). Supplementary 15. Figure S15: SEM images of MOF–MNRs after storing at room temperature for a week. Supplementary 16. Figure S16: SEM–EDS images of MOF–MNRs after performing reactions under 40°C for 12 h. Supplementary 17. Figure S17: (a) the XPS spectra of Pt[(C_6_H_5_)_3_P]_4_–MOF–MNRs and Pt[(C_6_H_5_)_3_P]_4_. (b) C 1 s spectrum of Pt[(C_6_H_5_)_3_P]_4_–MOF–MNRs and Pt[(C_6_H_5_)_3_P]_4_. Supplementary 18. Table S1: zeta potential of the MOF–MNRs and metal complex. Supplementary 19. Figure S18: molecular size of Pt[(C_6_H_5_)_3_P]_4_, C_10_H_14_O_4_Pt, and AlcDH.

## References

[1] Schwander T., von Borzyskowski L. S., Burgener S., Cortina N. S., Erb T. J. (2016). A synthetic pathway for the fixation of carbon dioxide in vitro. *Science*.

[2] Shi J. F., Wu Y. Z., Zhang S. H., Tian Y., Yang D., Jiang Z. (2018). Bioinspired construction of multi-enzyme catalytic systems. *Chemical Society Reviews*.

[3] Cao Y. F., Li X. Y., Xiong J. R., Wang L., Yan L. T., Ge J. (2019). Investigating the origin of high efficiency in confined multienzyme catalysis. *Nanoscale*.

[4] An H. D., Song J., Wang T. (2020). Metal–organic framework disintegrants: enzyme preparation platforms with boosted activity. *Angewandte Chemie International Edition*.

[5] Yang X., Chang J., Jiang Y., Xu Q., Wang M., Mao L. (2021). In vivo activation of pro-protein therapeutics via chemically engineered enzyme cascade reaction. *CCS Chemistry*.

[6] Rudroff F., Mihovilovic M. D., Groger H., Snajdrova R., Iding H., Bornscheuer U. T. (2018). Opportunities and challenges for combining chemo- and biocatalysis. *Nature Catalysis*.

[7] Li X. Y., Cao X., Xiong J. R., Ge J. (2019). Enzyme-metal hybrid catalysts for chemoenzymatic reactions. *Small*.

[8] Su H., Soldatov M. A., Roldugin V., Liu Q. (2022). Platinum single-atom catalyst with self-adjustable valence state for large- current-density acidic water oxidation. *eScience*.

[9] Chen W. H., Vazquez-Gonzalez M., Zoabi A., Abu-Reziq R., Willner I. (2018). Biocatalytic cascades driven by enzymes encapsulated in metal-organic framework nanoparticles. *Nature Catalysis*.

[10] Bornscheuer U. T., Huisman G. W., Kazlauskas R. J., Lutz S., Moore J. C., Robins K. (2012). Engineering the third wave of biocatalysis. *Nature*.

[11] Wang W., Qi X. X., Wu X. F. (2021). Palladium-catalyzed thiocarbonylation of benzyl chlorides with sulfonyl chlorides for the synthesis of arylacetyl thioesters. *Advanced Synthesis & Catalysis*.

[12] Cheng H. Y., Huo D., Zhu C. (2018). Combination cancer treatment through photothermally controlled release of selenous acid from gold nanocages. *Biomaterials*.

[13] Liang W. B., Xu H. S., Carraro F. (2019). Enhanced activity of enzymes encapsulated in hydrophilic metal–organic frameworks. *Journal of the American Chemical Society*.

[14] Makkee M., Kieboom A., Vanbekkum H. (1980). Combined action of enzyme and metal catalyst, applied to the preparation of D-mannitol. *Journal of the Chemical Society, Chemical Communications*.

[15] Li X. Y., Cao Y. F., Luo K. (2019). Highly active enzyme-metal nanohybrids synthesized in protein-polymer conjugates. *Nature Catalysis*.

[16] Zhang Y., Ge J., Liu Z. (2015). Enhanced activity of immobilized or chemically modified enzymes. *ACS Catalysis*.

[17] Ge J., Lei J. D., Zare R. N. (2012). Protein-inorganic hybrid nanoflowers. *Nature Nanotechnology*.

[18] Cortes-Clerget M., Akporji N., Zhou J. G. (2019). Bridging the gap between transition metal- and bio-catalysis via aqueous micellar catalysis. *Nature Communications*.

[19] Rifaie-Graham O., Galensowske N. F., Dean C. (2021). Shear stress-responsive polymersome nanoreactors inspired by the marine bioluminescence of dinoflagellates. *Angewandte Chemie International Edition*.

[20] Himiyama T., Waki M., Maegawa Y., Inagaki S. (2019). Cooperative catalysis of an alcohol dehydrogenase and rhodium-modified periodic mesoporous organosilica. *Angewandte Chemie International Edition*.

[21] Jiao L., Wang J., Jiang H. (2021). Microenvironment modulation in metal−organic framework-based catalysis. *Accounts of Materials Research*.

[22] Gao S. Q., Wang Z. H., Ma L., Liu Y., Gao J., Jiang Y. (2020). Mesoporous core-shell nanostructures bridging metal and biocatalyst for highly efficient cascade reactions. *ACS Catalysis*.

[23] Liu C., Sun Q., Lin L. (2020). Ternary MOF-on-MOF heterostructures with controllable architectural and compositional complexity via multiple selective assembly. *Nature Communications*.

[24] Jiao L., Seow J. Y., Skinner W. S., Wang Z. U., Jiang H. L. (2019). Metal-organic frameworks: structures and functional applications. *Materials Today*.

[25] Wei Y. S., Zhang M., Zou R. Q., Xu Q. (2020). Metal–organic framework-based catalysts with single metal sites. *Chemical Reviews*.

[26] Chen L. Y., Xu Q. (2019). Metal-organic framework composites for catalysis. *Matter*.

[27] Ding J., Tang Y., Zheng S. (2022). The synthesis of MOF derived carbon and its application in water treatment. *Nano Research*.

[28] Li H., Shi L. F., Li C., Fu X., Huang Q., Zhang B. (2020). Metal–organic framework based on *α*-cyclodextrin gives high ethylene gas adsorption capacity and storage stability. *ACS Applied Materials & Interfaces*.

[29] Liang L. F., Liu C. P., Jiang F. L. (2017). Carbon dioxide capture and conversion by an acid-base resistant metal- organic framework. *Nature Communications*.

[30] Farha O. K., Yazaydin A. O., Eryazici I. (2010). *De novo* synthesis of a metal -organic framework material featuring ultrahigh surface area and gas storage capacities. *Nature Chemistry*.

[31] Li F., Liu C., Yuan D. (2022). Ultrahigh hydrogen uptake in an interpenetrated Zn_4_O-based metal–organic framework. *CCS Chemistry*.

[32] Wang C., An B., Lin W. B. (2019). Metal–organic frameworks in solid-gas phase catalysis. *ACS Catalysis*.

[33] Jiao L., Wang Y., Jiang H. L., Xu Q. (2018). Metal-organic frameworks as platforms for catalytic applications. *Advanced Materials*.

[34] Katz M. J., Mondloch J. E., Totten R. K. (2014). Simple and compelling biomimetic metal–organic framework catalyst for the degradation of nerve agent simulants. *Angewandte Chemie International Edition*.

[35] Tu Y., Li H., Tu T., Zhang Q. (2022). Lamellar enzyme-metal–organic framework composites enable catalysis on large substrates. *CCS Chemistry*.

[36] Hu X., Li Z., Xue H., Huang X., Cao R., Liu T. F. (2020). Designing a bifunctional Brønsted acid–base heterogeneous catalyst through precise installation of ligands on metal–organic frameworks. *CCS Chemistry*.

[37] Hou J., Zhang H. C., Simon G. P., Wang H. T. (2019). Polycrystalline advanced microporous framework membranes for efficient separation of small molecules and ions. *Advanced Materials*.

[38] He L. C., Brasino M., Mao C. C. (2017). DNA-assembled core-satellite upconverting-metal-organic framework nanoparticle superstructures for efficient photodynamic therapy. *Small*.

[39] Zheng G., Pastoriza-Santos I., Pérez-Juste J., Liz-Marzán L. M. (2021). Plasmonic metal-organic frameworks. *SmartMat*.

[40] Liu Z., Zheng F., Xiong W., Li X., Yuan A., Pang H. (2021). Strategies to improve electrochemical performances of pristine metal-organic frameworks-based electrodes for lithium/sodium-ion batteries. *SmartMat*.

[41] Liu C., Bai Y., Li W., Yang F., Zhang G., Pang H. (2022). In situ growth of three-dimensional MXene/metal-organic framework composites for high-performance supercapacitors. *Angewandte Chemie International Edition*.

[42] Chen D. X., Yang W. J., Jiao L., Li L., Yu S. H., Jiang H. L. (2020). Boosting catalysis of Pd nanoparticles in MOFs by pore wall engineering: the roles of electron transfer and adsorption energy. *Advanced Materials*.

[43] Cai G., Ding M., Wu Q., Jiang H. (2020). Encapsulating soluble active species into hollow crystalline porous capsules beyond integration of homogeneous and heterogeneous catalysis. *National Science Review*.

[44] Denard C. A., Hartwig J. F., Zhao H. M. (2013). Multistep one-pot reactions combining biocatalysts and chemical catalysts for asymmetric synthesis. *ACS Catalysis*.

[45] Wang Y. J., Zhao H. M. (2016). Tandem reactions combining biocatalysts and chemical catalysts for asymmetric synthesis. *Catalysts*.

[46] Wang Z. J., Clary K. N., Bergman R. G., Raymond K. N., Toste F. D. (2013). A supramolecular approach to combining enzymatic and transition metal catalysis. *Nature Chemistry*.

[47] Maughan R. (1982). A simple, rapid method for the determination of glucose, lactate, pyruvate, alanine, 3-hydroxybutyrate and acetoacetate on a single 20-*μ*l blood sample. *Clinica Chimica Acta*.

[48] Xu Z. L., Xiao G. W., Li H. F. (2018). Compartmentalization within self-assembled metal–organic framework nanoparticles for tandem reactions. *Advanced Functional Materials*.

[49] Zhang J., Jin N., Ji N. (2021). The encounter of biomolecules in metal–organic framework micro/nano reactors. *ACS Applied Materials & Interfaces*.

[50] Zhang W. L., Shi W. X., Ji W. L. (2020). Microenvironment of MOF channel coordination with Pt NPs for selective hydrogenation of unsaturated aldehydes. *ACS Catalysis*.

[51] Sharp C. H., Bukowski B. C., Li H. Y. (2021). Nanoconfinement and mass transport in metal-organic frameworks. *Chemical Society Reviews*.

[52] Meng F., Zhang S., Ma L. (2018). Construction of hierarchically porous nanoparticles@metal-organic frameworks composites by inherent defects for the enhancement of catalytic efficiency. *Advanced Materials*.

[53] Shen Y., Pan T., Wu P. (2021). Regulating electronic status of platinum nanoparticles by metal–organic frameworks for selective catalysis. *CCS Chemistry*.

[54] Schaate A., Roy P., Godt A. (2011). Modulated synthesis of Zr-based metal–organic frameworks: from nano to single crystals. *Chemistry - A European Journal*.

[55] Lohse M., Stassin T., Naudin G. (2016). Sequential pore wall modification in a covalent organic framework for application in lactic acid adsorption. *Chemistry of Materials*.

