# Supplementary material for: Chemo-Biocascade Reactions Enabled by Metal–Organic Framework Micro-Nanoreactor
Source: Research (Wash D C). 2022 Aug 15;2022:9847698. doi: 10.34133/2022/9847698 (PMC9414180; doi:10.34133/2022/9847698)
Supplement: Supplementary Materials — Supplementary 1. Figure S1: BET comparison of UiO-66-NH2 before and after hydrophobic modification. Supplementary 2. Figure S2: X-ray diffraction data for the synthesized, modified, and assembled MOF–MNRs. Supplementary 3. Figure S3: SEM images of MOF–MNRs. Supplementary 4. Figure S4: Fluorescence images of MOF–MNRs entrapped with FITC labelled AlcDH and RB labeled NAD+. Supplementary 5. Figure S5: comparison of the catalytic efficiency of the whole cycle reaction and the two half reaction catalyzed by either metal complex or AlcDH. Supplementary 6. Recyclability of AlcDH/NAD + @MOF–MNRs for concurrent chemo-biocatalysis in the reduction of pyruvic acid. Supplementary 7. Figure S7: SEM images of PMMA–MNRs obtained in the presence of surfactants. Supplementary 8. Figure S8: comparison of enzyme activity of AlcDH/NAD + @MOF–MNRs and AlcDH/NAD + @PMMA–MNRs after 12 h. Supplementary 9. Figure S9: Diffusion investigation of FITC labelled AlcDH inside MOF–MNRs and PMMA–MNRs. Supplementary 10. Figure S10: comparison of the fluorescence intensity of the generated NADH after exchanging the location of AlcDH and metal complex and in the presence of protease. Supplementary 11. Figure S11: comparison of the UV/Vis absorption of AlcDH between unencapsulated AlcDH/NAD+, AlcDH/NAD + @PMMA–MNRs, and AlcDH/NAD + @MOF–MNRs after the reactions. Supplementary 12. Figure S12: UV/Vis absorption of pure AlcDH within 50 h. Supplementary 13. Figure S13: UV/Vis absorption of AlcDH/NAD + @MOF–MNRs within 8 d. Supplementary 14. Figure S14: activities of the concurrent chemo-bioreactions of pyruvic acid to lactic acid immediately after fresh preparation of the catalysts (red line) and after storage together at room temperature for a time interval of one week (black line). Supplementary 15. Figure S15: SEM images of MOF–MNRs after storing at room temperature for a week. Supplementary 16. Figure S16: SEM–EDS images of MOF–MNRs after performing reactions under 40°C for 12 h. Supplementary 17. Figu [file 9847698.f1.docx]

Supplementary Materials

**Chemo–Bio Cascade Reactions Enabled by Metal–Organic Framework Micro–nano Reactor**

Jing Zhang^†,1^ Yu Shen^†,1^ Na Jin^†,1^ Xiaopeng Zhao,^1^ Hongfeng Li,^1^ Ning Ji,^1^ Yingjie Li,^1^ Baoli Zha,^1^ Lin Li,^1,2^ Xikuang Yao,^1^ Suoying Zhang,^1^ Fengwei Huo,^1^ and Weina Zhang*^,1^

^1^Key Laboratory of Flexible Electronics (KLOFE), Institute of Advanced Materials (IAM), Nanjing Tech University (NanjingTech), Nanjing 211800, China

^2^The Institute of Flexible Electronics (IFE, Future Technologies), Xiamen University, Xiamen 361005, Fujian, China

^⋆^Correspondence should be addressed to Weina Zhang; iamwnzhang@njtech.edu.cn

**Table of Contents**

[**Characterization of different MNRs or UiO-66-NH_2_ NPs** 3](#_Toc102085690)

[**Encapsulation of enzyme inside MOF–MNRs** 5](#_Toc102085691)

[**Encapsulation of AlcDH inside MOF–MNRs** 6](#_Toc102085692)

[**Characterization of PMMA–MNRs** 8](#_Toc102085693)

[**AlcDH@MOF–MNRs for protection and compartmentalization** 11](#_Toc102085694)

[**Stability of AlcDH@MOF–MNRs compared with other systems** 12](#_Toc102085695)

[**Interaction between metal complexes and MOF–MNRs** 15](#_Toc102085696)

[**Molecular structures** 15](#_Toc102085696)

**Result and discussion**

**Characterization of different MNRs or UiO-66-NH_2_ NPs**


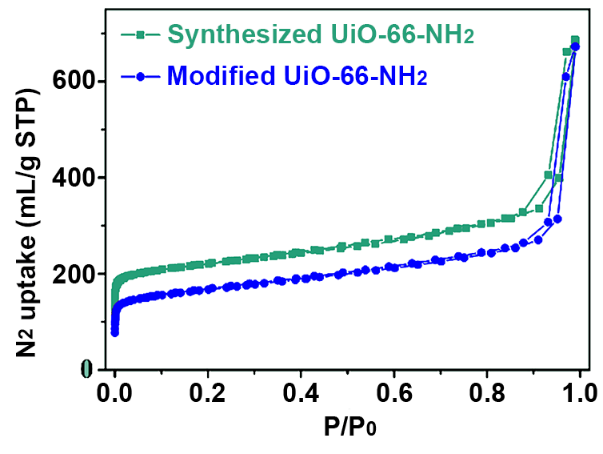


Figure S1. Nitrogen adsorption–desorption curves of UiO-66-NH_2_ before and after hydrophobic modification.


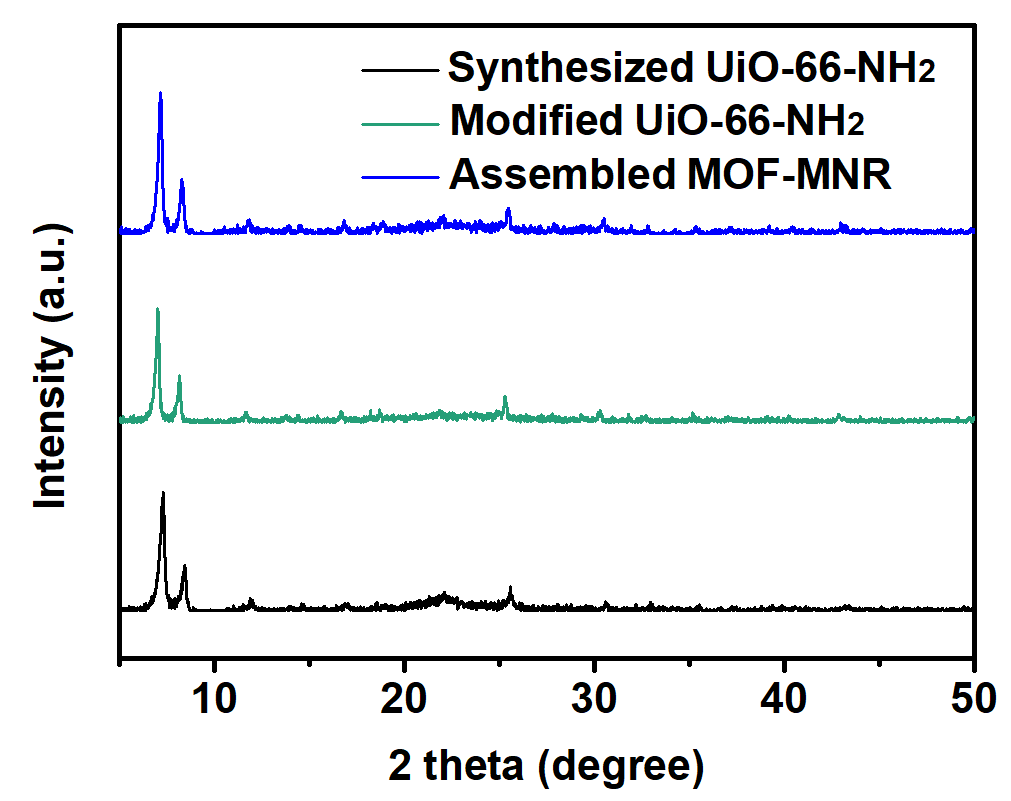


Figure S2. X-ray diffraction data for the synthesized, modified and assembled MOF–MNRs.


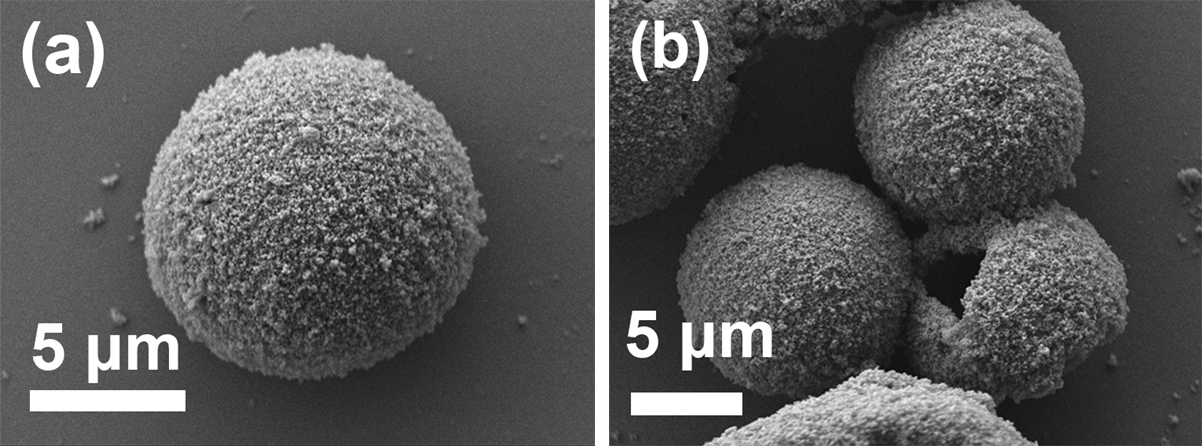


Figure S3. SEM images of MOF–MNRs. (a) Single MOF–MNR. (b) Broken MOF–MNRs.

**Encapsulation of enzyme inside MOF–MNRs**


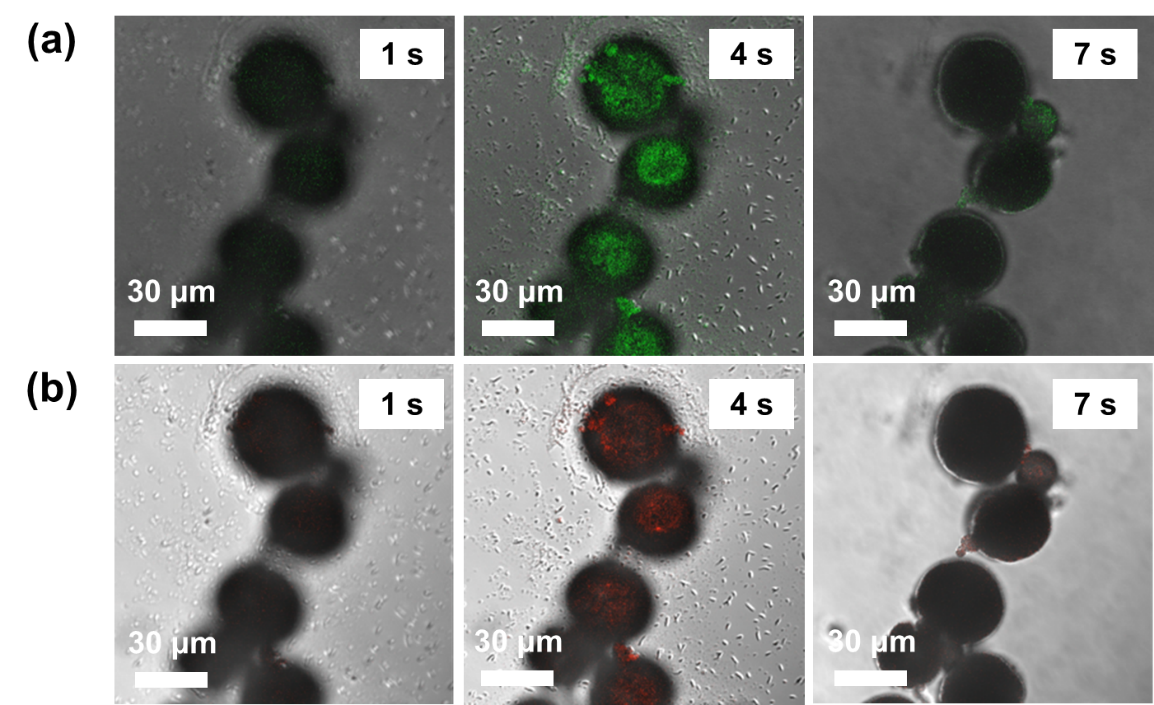


Figure S4. Fluorescence images of MOF–MNRs entrapped with FITC labelled AlcDH and RB labeled NAD^+^. (a) Asymptotic process of scanning along the z-axis of the FITC labelled AlcDH@MOF–MNRs. (b) Asymptotic process of scanning along the z-axis of the RB labeled NAD^+^@MOF–MNRs. The excitation wavelengths are 488 nm and 543 nm.

**Encapsulation of AlcDH inside MOF–MNRs**


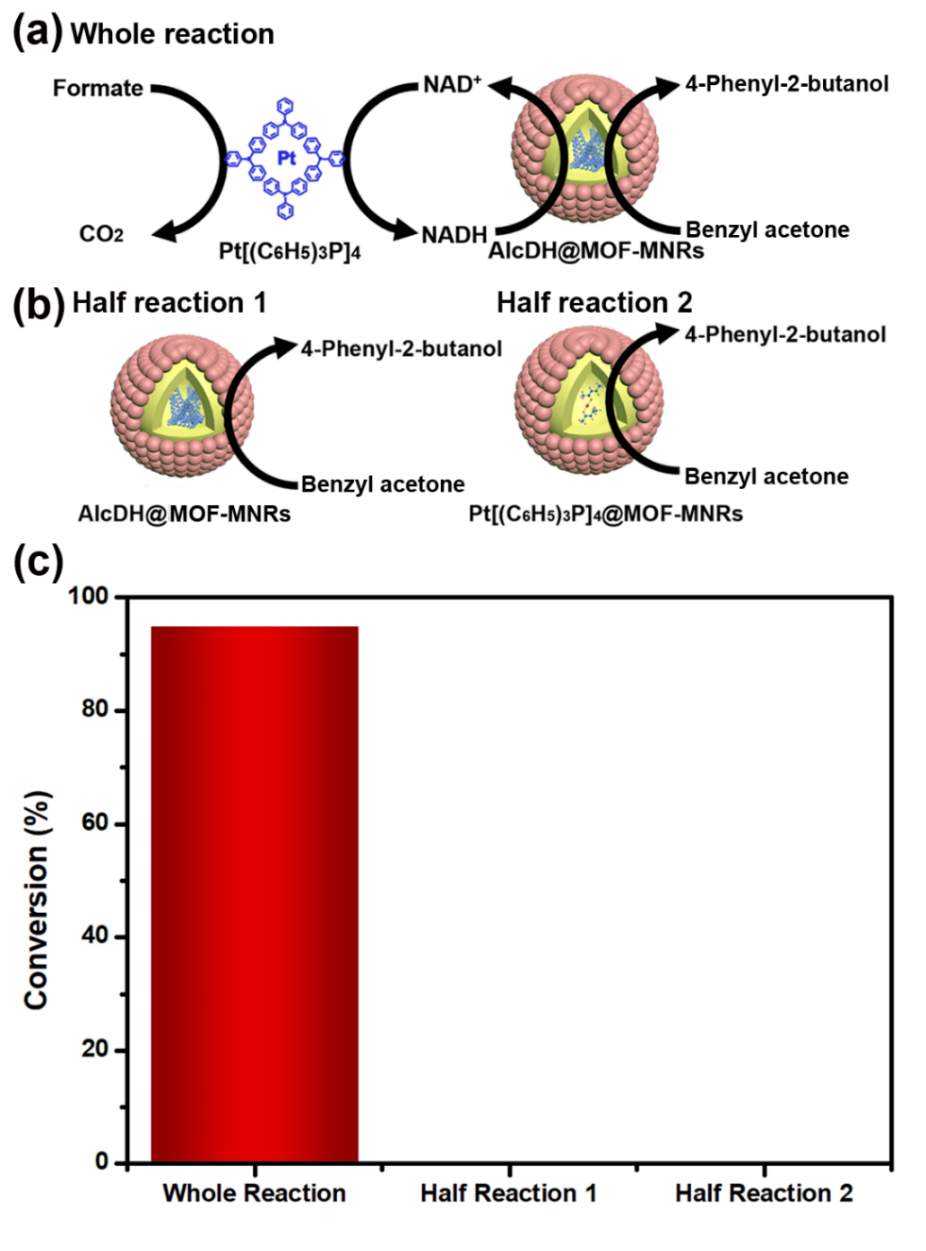


Figure S5. Comparison of the catalytic efficiency of the whole cycle reaction and the two half reaction catalyzed by either metal complex or AlcDH. (a) Schematic illustration about the reduction of benzyl acetone catalyzed by AlcDH@MOF–MNRs with Pt[(C_6_H_5_)_3_P]_4_. (b) Schematic illustration about the reduction of benzyl acetone catalyzed by AlcDH@MOF–MNRs (left) and schematic illustration about the reduction of benzyl acetone catalyzed by Pt[(C_6_H_5_)_3_P]_4_@MOF–MNRs (right). (c) Catalytic results of the whole chemo–bio concurrent reactions compared with the above two reactions with only AlcDH or Pt[(C_6_H_5_)_3_P]_4_ inside the MNRs.





Figure S6. Recyclability of AlcDH@MOF–MNRs for concurrent chemo–bio catalysis in the reduction of pyruvic acid.

**Characterization of PMMA–MNRs**


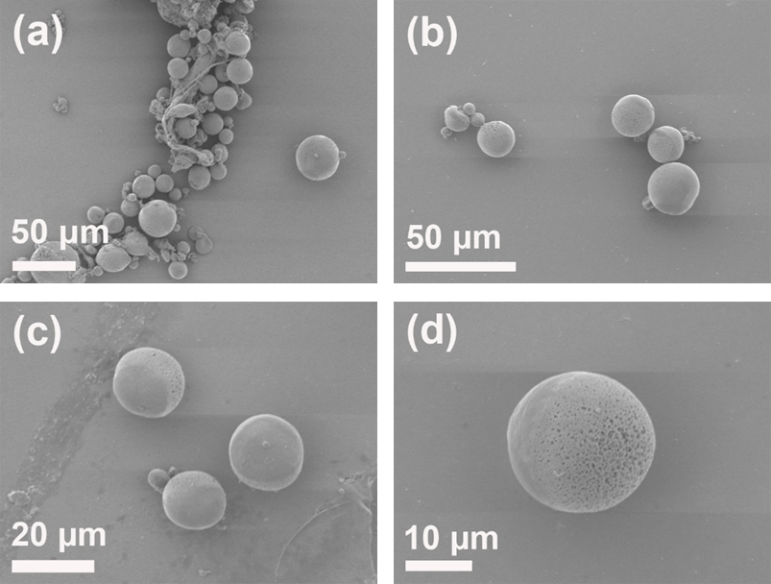


Figure S7. SEM images of PMMA–MNRs obtained in the presence of surfactants. (a, b, c) PMMA–MNRs obtained in the presence of Span 80 with 3% polymer. (d) Single PMMA–MNR.


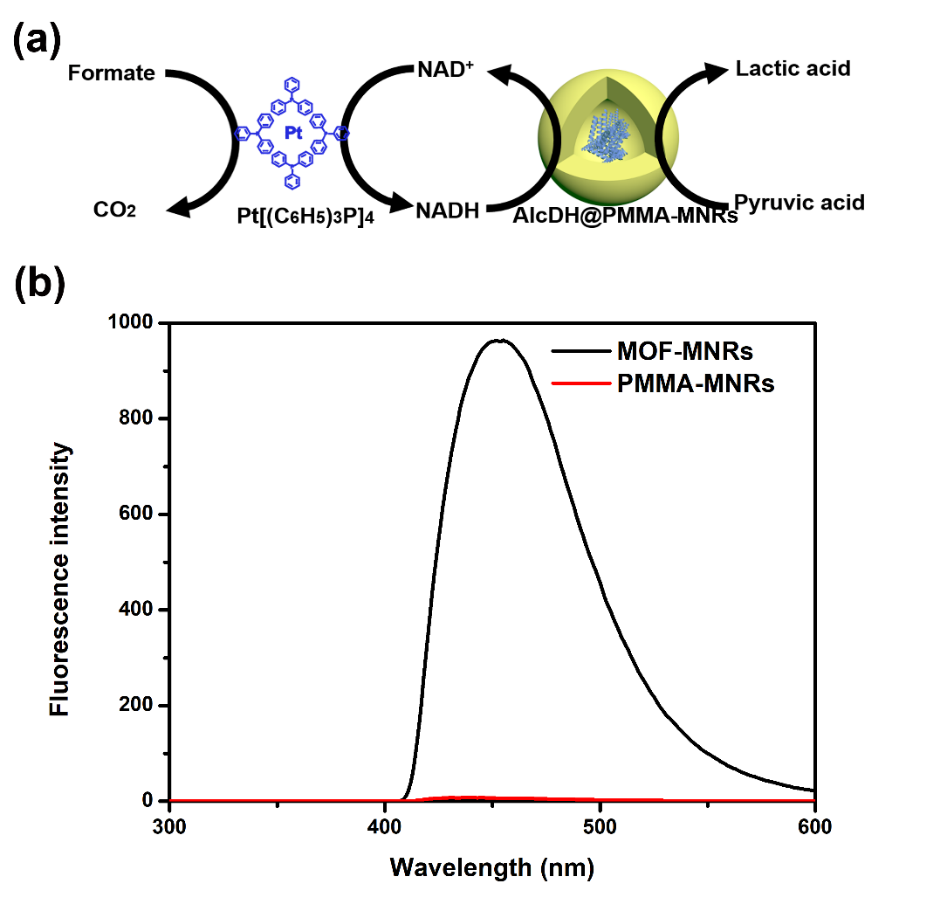


Figure S8. Comparison of enzyme activity of AlcDH@MOF–MNRs and AlcDH@PMMA–MNRs after 12 h.





Figure S9. Diffusion investigation of FITC labelled AlcDH inside MOF–MNRs and PMMA–MNRs. Fluorescence intensity of the FITC labelled AlcDH which have been released into ethanol solution from AlcDH@PMMA–MNRs and AlcDH@MOF–MNRs.

**AlcDH@MOF–MNRs for protection and compartmentalization**


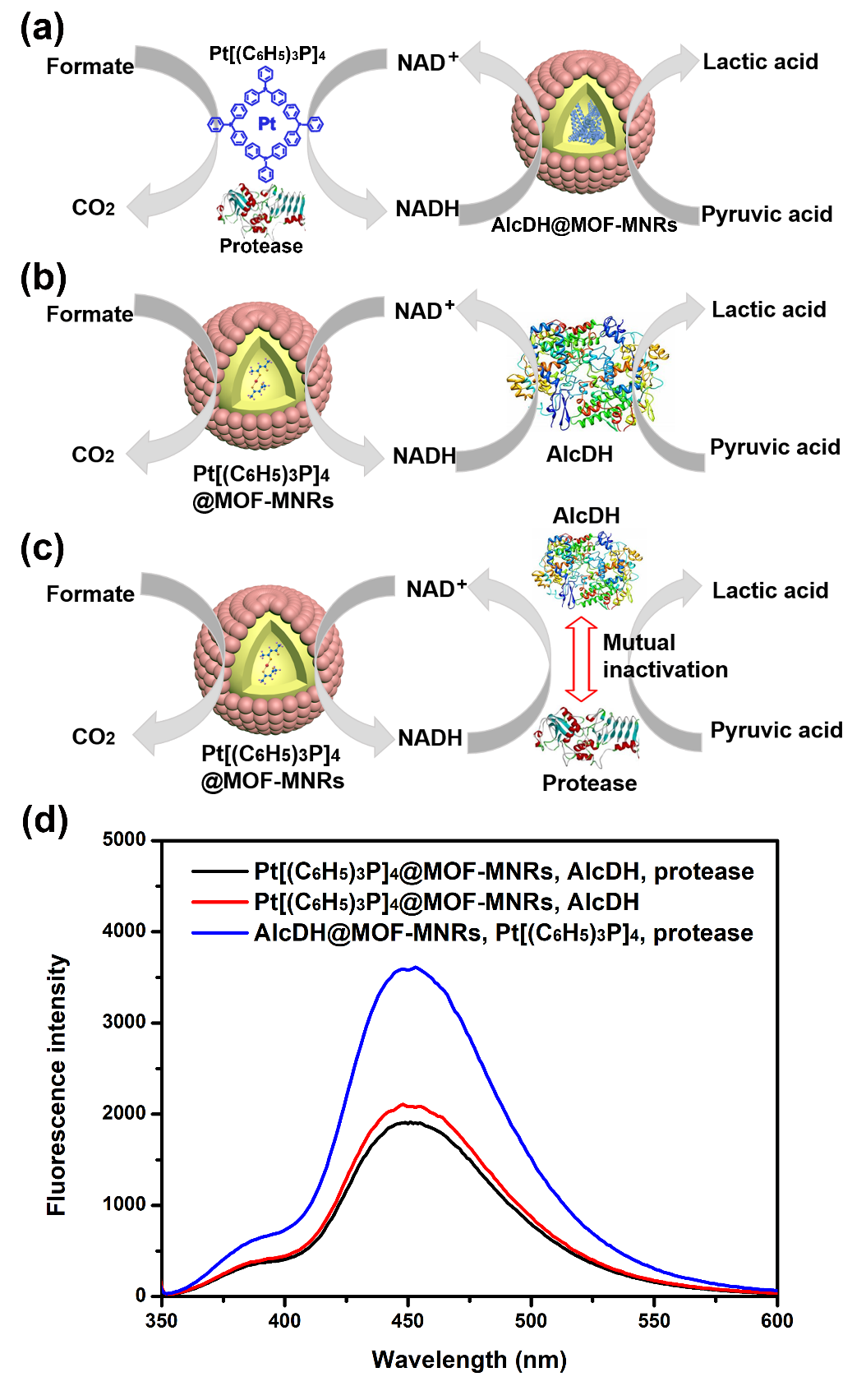


Figure S10. Comparison of the fluorescence intensity of the generated NADH after exchanging the location of AlcDH and metal complex, and in the presence of protease.

**Stability of AlcDH@MOF–MNRs compared with other systems**





Figure S11. Comparison of the UV/Vis absorption of AlcDH between unencapsulated AlcDH, AlcDH@PMMA–MNRs and AlcDH@MOF–MNRs after the reactions.


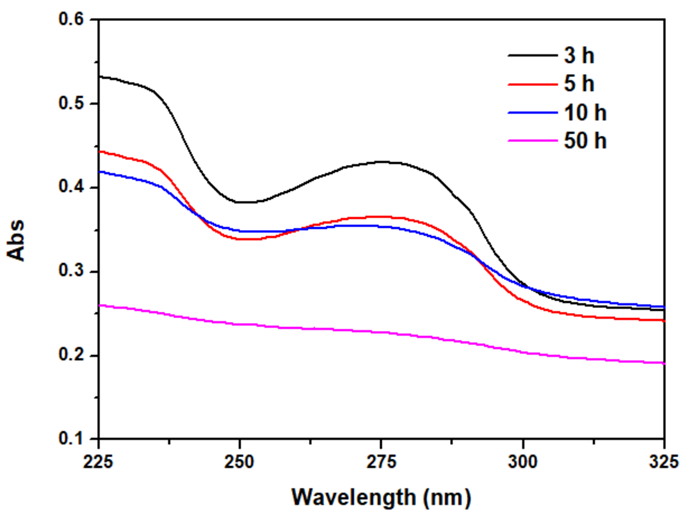


Figure S12. UV/Vis absorption of pure AlcDH within 50 h.





Figure S13. UV/Vis absorption of AlcDH@MOF–MNRs within 8 d.


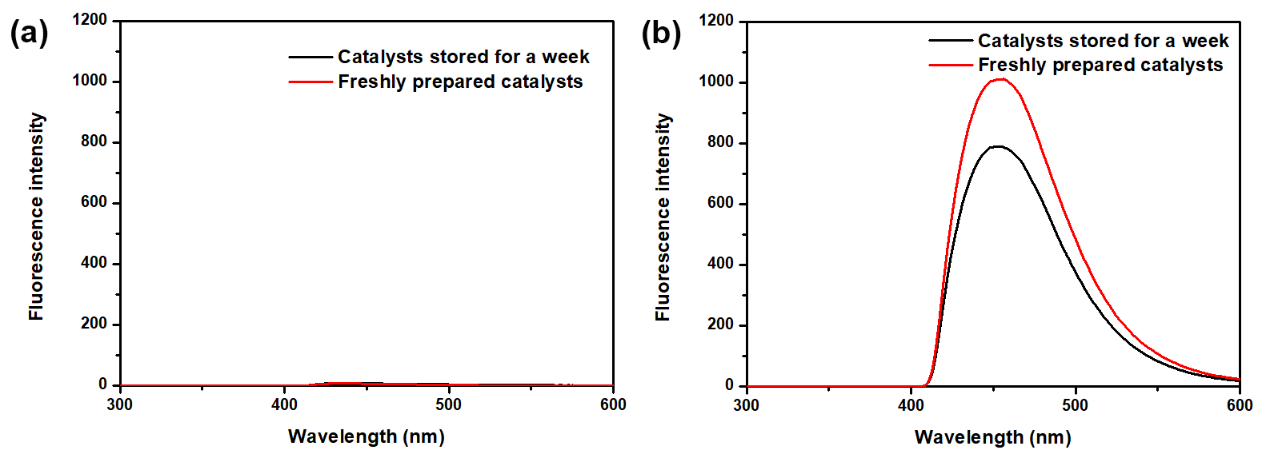


Figure S14. Activities of the concurrent chemo–bio reactions of pyruvic acid to lactic acid immediately after fresh preparation of the catalysts (red line) and after storage together at room temperature for a time-interval of one week (black line). (a) Comparison of enzyme activities in the concurrent chemo–bio reactions of pyruvic acid to lactic acid between freshly prepared enzymes and enzymes which have been stored at room temperature for a week. (b) Comparison of enzyme activities in the concurrent chemo–bio reactions of pyruvic acid to lactic acid between freshly prepared AlcDH@MOF–MNRs and AlcDH@MOF–MNRs which have been stored at room temperature for a week.


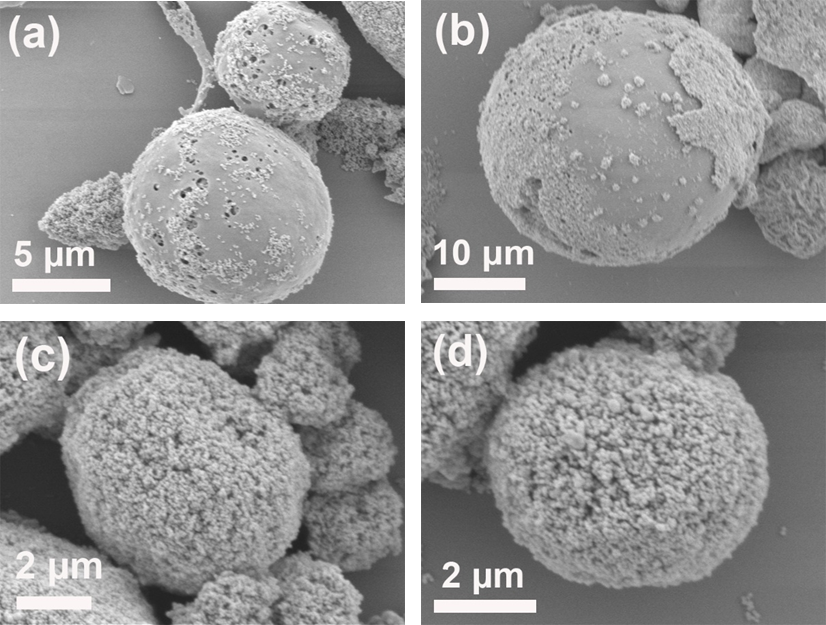


Figure S15. SEM images of MOF–MNRs after storing at room temperature for a week.

**Interaction between metal complexes and MOF–MNRs**


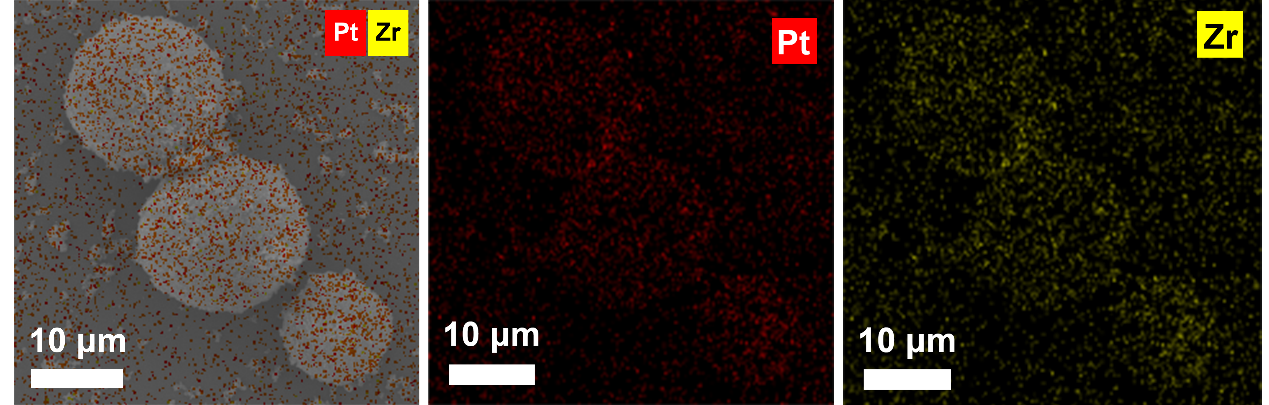


Figure S16. SEM–EDS images of MOF–MNRs after performing reactions under 40 °C for 12 h.

**
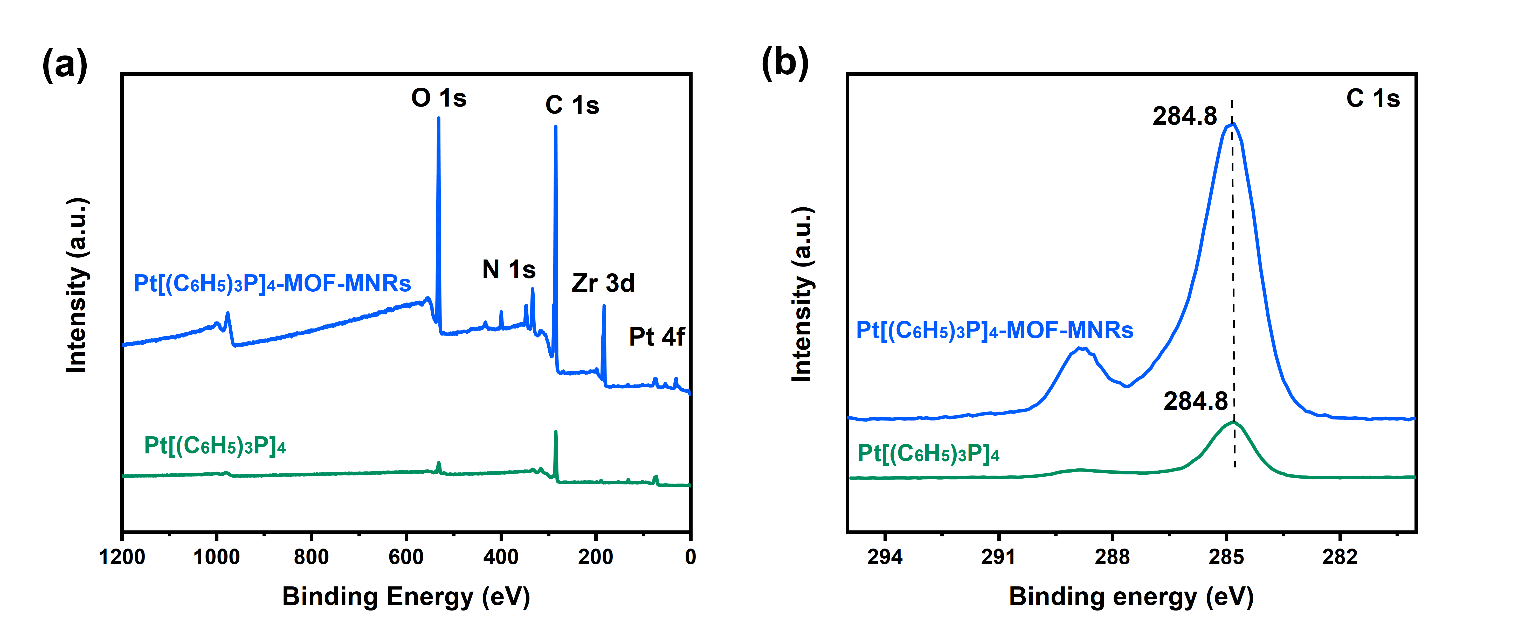
**

Figure S17. (a) The XPS spectra of Pt[(C_6_H_5_)_3_P]_4_–MOF–MNRs and Pt[(C_6_H_5_)_3_P]_4_. (b) C 1s spectrum of Pt[(C_6_H_5_)_3_P]_4_–MOF–MNRs and Pt[(C_6_H_5_)_3_P]_4_.

Table S1. Zeta potential of the MOF–MNRs, PMMA–MNRs and metal complex.

| Samples | Zeta potential (mV) |
| --- | --- |
| MOF–MNRs | 1.65 |
| PMMA–MNRs | –4.23 |
| Metal complex | –5.87 |

**Molecular structures**


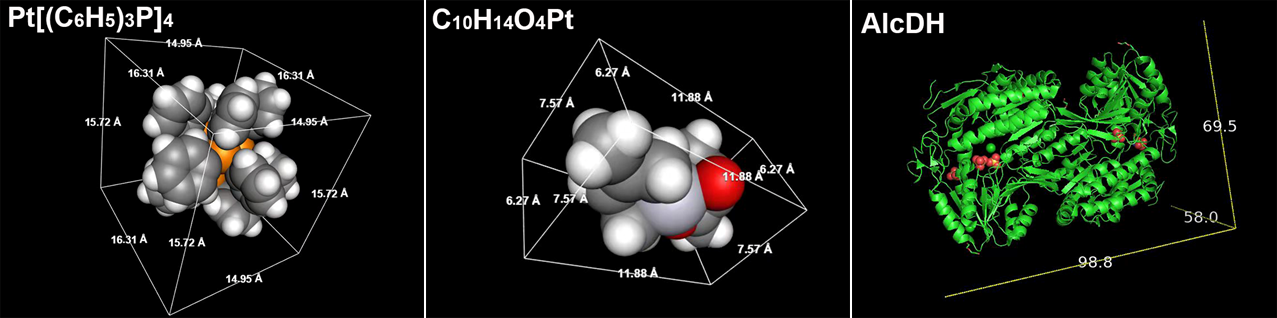


Figure S18. Molecular size of Pt[(C_6_H_5_)_3_P]_4_, C_10_H_14_O_4_Pt and AlcDH.
